# Early prediction of infarct size by ultra-fast online assessment of systolic left ventricular longitudinal function

**DOI:** 10.1186/1532-429X-14-S1-P260

**Published:** 2012-02-01

**Authors:** Sebastian Buss, Grigorios Korosoglou, Florian Andre, Evangelos Giannitsis

**Affiliations:** 1 Department of Cardiology, University of Heidelberg, Heidelberg, Germany

## Summary

TMAD provides the possibility for the assessment of global systolic longitudinal function in patients with STEMI and correlates well with the extent of myocardial infarction, determined by LGE-CMR. TMAD is a novel, ultra-fast, sensitive and easily reproducible parameter and therefore a valuable predictor for the extent of myocardial infarction. Further prospective studies have to be conducted to test TMAD as a promising tool for clinical routine.

## Background

In clinical routine, fast assessment of infarct size is crucial for accurate early risk assessment. Global left ventricular (LV) longitudinal strain is associated with infarct size and outcome. The measurement of global longitudinal function is often dependent on image quality, experience of the observer and it is time-consuming. We investigated global systolic LV longitudinal function with TMAD (tissue motion annular displacement), a novel method based on a 2-D strain tissue tracking algorithm, and compared it with contrast-enhanced magnetic resonance imaging (CMR). The aim of the study was to investigate whether TMAD is able to diagnose LV infarct size early after successful revascularization in patients with first acute ST-segment elevation myocardial infarction (STEMI).

## Methods

Twenty-one patients underwent CMR and trans-thoracic echocardiography 3 ± 1 day after successfully reperfused STEMI. On a whole-body 1.5T 32 channel MRI system (Philips Achieva), vector ECG-gated short axis, 2-3-4 chamber SSFP cine images as well as late gadolinium enhancement CMR (LGE-CMR) in similar planes were acquired using a standard inversion-recovery sequence. TMAD was measured by standard transthoracic echocardiography (GE vivid 7000). Serological markers for infarction (Troponin, creatine kinase) were measured from plasma samples.

## Results

There was a high correlation for the percentage of LGE-CMR infarcted LV (r=0.63, p<0.05) as well as absolute LV infarct mass (r=0.71, p<0.001) with TMAD. TMAD also showed a significant correlation with serological markers (creatine kinase: r=0.52, p<0.05 and cTNT: r=0.50, p<0.05). Time for analysis of TMAD was less than 15sec. The intra- and inter-observer variability for TMAD was very low (1.4 ± 1.1 % and 1.8 ± 1.3 %).

## Conclusions

TMAD provides the possibility for the assessment of global systolic longitudinal function in patients with STEMI and correlates well with the extent of myocardial infarction, determined by LGE-CMR. TMAD is a novel, ultra-fast, sensitive and easily reproducible parameter and therefore a valuable predictor for the extent of myocardial infarction. Further prospective studies have to be conducted to test TMAD as a promising tool for clinical routine.

## Funding

No funding.

